# The role of social intelligence and creativity as personal resources for coping with uncertainty during primary career self-determination in late adolescence

**DOI:** 10.1192/j.eurpsy.2022.852

**Published:** 2022-09-01

**Authors:** O. Chesnokova, S. Churbanova, S. Molchanov, O. Markish

**Affiliations:** Lomonosov Moscow State University, Developmental Psychology Department, Moscow, Russian Federation

**Keywords:** adolescence, Stress, social intelligence, creativity

## Abstract

**Introduction:**

Social intelligence (SI) and creativity (Cr) are resources to develop adaptive coping strategies and positive emotions that can help combat feelings of fragility and vulnerability caused by uncertainty during primary career self-determination as the developmental task in late adolescence.

**Objectives:**

The aim of the study was to compare SI and Cr of students with high and low level of tolerance for uncertainty (TU) during two last years in the middle school.

**Methods:**

Participants were 200 students (15-17 years old). The level of TU was estimated by Budner’s scale (Kornilova, 2010). Social intelligence was assessed by O’Sullivan & Guilford’s Tests, creativity was measured by CAP. In addition, we estimate career adapt-abilities applied to present and prospective future career decisions.

**Results:**

Two contrast clusters based on TU level were analysed. The level of SI and Cr were various within each group. There appears to be an association between TU and career adapt-abilities (at r ∼ .37, p < .05). Mostly female students with low level of TU and above average scores on SI and Cr demonstrate the effective coping strategy dealing with stress (at r ∼ .45, p < .01) emotionally focus on nearest professional future, seek social support. Coping strategy of students with high TU, SI and Cr is focusing on nearest and distant professional future, on task-oriented content and the social status of the future profession. They are open to new career experience and flexibility in the use of future professional skills (at r ∼ .56, p < .001).

**Conclusions:**

Employing their SI and Cr give new opportunities to understand and prevent the development of stress and provide age-specific support to prospective students during primary career self-determination.

**Disclosure:**

Research is supported by the Russian Humanitarian Research Foundation, project No. 18-013-01067.

